# Supplementary material for: Laparoscopic Right Hemihepatectomy after Future Liver Remnant Modulation: A Single Surgeon’s Experience
Source: Cancers (Basel). 2023 May 21;15(10):2851. doi: 10.3390/cancers15102851 (PMC10216226; doi:10.3390/cancers15102851)
Supplement: Supplementary file 1 [file cancers-15-02851-s001.zip › cancers-2371657-supplementary.pdf]

Supplementary Table S1. Univariable and Multivariable Regression Analyses

	TOLS				Pringle Duration				Operative Time			
	**Univariable**		**Multivariable**		**Univariable**		**Multivariable**		**Univariable**		**Multivariable**	
Term	OR (95% CI)	P	aOR (95% CI)	P	OR (95% CI)	P	aOR (95% CI)	P	OR (95% CI)	P	aOR (95% CI)	P
BMI	0.916 (0.681 - 1.179)	0.516	-	-	2.183 (0.688 - 6.928)	0.177	-	-	0.382 (2.114e-4 - 6.896e+2)	0.795	-	-
Cirrhosis	1.467 (0.190 - 30.363)	0.747	-	-	101.291 (2.880e-04 - 3.562e+07)	0.470	-	-	3.884e+36 (1.462e-03 - 1.034e+76)	**0.068**	2.872e+29 (0.001 - 1.034e+76)	0.197
Previous Liver Surgery	0.264 (0.065 - 1.004)	**0.053**	0.134 (0.065 - 1.004)	0.117	32.427 (6.340e-03 - 1.657e+05)	0.416	-	-	4.027e+22 (1.273e-2 - 1.274e+47)	**0.070**	1.705e+18 (0.013 - 1.274e+47)	0.250
Size largest lesion	0.991 (0.976 - 1.006)	**0.098**	0.986 (0.976 - 1.006)	0.147	1.002 (0.909 - 1.105)	0.961	-	-	1.647 (0.926 - 2.927)	**0.088**	1.468 (0.926 - 2.927)	0.192
No of Lesions	0.735 (0.499 - 1.057)	**0.099**	0.707 (0.499 - 1.057)	0.186	1.098 (0.067 - 17.997)	0.947	-	-	21057.25 (0.003 - 1.329e+11)	0.207	-	-
FLRM	1.198 (0.305 - 4.436)	0.788	5.076 (0.305 - 4.436)	0.183	34440.9 (8.944 - 1.327e+08)	**0.014**	-	**-**	3.706e+31 (4.476e+10 - 3.052e+52)	**0.003**	2.952e+18 (4.476e+10 - 3.052e+52)	0.222
